# Behavioural Syndrome in a Solitary Predator Is Independent of Body Size and Growth Rate

**DOI:** 10.1371/journal.pone.0031619

**Published:** 2012-02-20

**Authors:** Marina J. Nyqvist, Rodolphe E. Gozlan, Julien Cucherousset, J. Robert Britton

**Affiliations:** 1 Centre for Conservation Ecology & Environmental Sciences, School of Applied Sciences, Bournemouth University, Poole, United Kingdom; 2 CNRS, UPS, ENFA; UMR5174 EDB (Laboratoire Évolution et Diversité Biologique), Toulouse, France; 3 Université de Toulouse; UPS; UMR5174 EDB; Toulouse, France; Macquarie University, Australia

## Abstract

Models explaining behavioural syndromes often focus on state-dependency, linking behavioural variation to individual differences in other phenotypic features. Empirical studies are, however, rare. Here, we tested for a size and growth-dependent stable behavioural syndrome in the juvenile-stages of a solitary apex predator (pike, *Esox lucius*), shown as repeatable foraging behaviour across risk. Pike swimming activity, latency to prey attack, number of successful and unsuccessful prey attacks was measured during the presence/absence of visual contact with a competitor or predator. Foraging behaviour across risks was considered an appropriate indicator of boldness in this solitary predator where a trade-off between foraging behaviour and threat avoidance has been reported. Support was found for a behavioural syndrome, where the rank order differences in the foraging behaviour between individuals were maintained across time and risk situation. However, individual behaviour was independent of body size and growth in conditions of high food availability, showing no evidence to support the state-dependent personality hypothesis. The importance of a combination of spatial and temporal environmental variation for generating growth differences is highlighted.

## Introduction

Empirical studies across a range of animal taxa are increasingly demonstrating the existence of personalities, where individuals within populations vary consistently in their behaviour over time [1]–[3]. When individual behaviours are consistent or co-vary across situations or contexts, where a context is a functional behavioural category (e.g. feeding, mating, predator avoidance or dispersal), and a situation is the set of conditions at a particular time which can involve different levels along an environmental gradient (e.g. foraging behaviours in different habitats), it is referred to as a behavioural syndrome [2], [4], [5]. Although individual consistency of single behaviours is considered to contribute meaningfully to the stability of the behavioural syndrome they comprise [6]–[8], repeated observations of individuals over time within situations or contexts are lacking in many studies [5], [9], [10]. Despite this, and in conjunction with inconsistent methodologies employed to assess behavioural traits [11], [12], behavioural syndromes are considered to be widespread [2]. Furthermore, a focus on characterising behavioural syndromes in social or territorial species that show parental care or build nests, exhibit dominance hierarchies or other social structures, such as shoaling [11], [13] has resulted in a paucity of studies in other species, such as in solitary apex predators. Yet characterizing behavioural syndromes in ecologically-different species with contrasting behavioural life-histories should improve our understanding of the extent of behavioural syndromes and their ecological importance. For example, identifying behavioural syndromes in an apex predator may be particularly important for understanding their effect on trophic interactions and influence on prey fish communities [14], [15].

Behavioural syndromes are temporally stable when the same association between different behaviours occurs at different stages in time [4], [16]. Temporal stability in behavioural syndromes suggests that individual behaviours may not be able to evolve independently and are therefore considered to be of particular evolutionary significance [4], [17]. Exploring the mechanisms involved in maintaining behavioural syndromes in animals has therefore recently received considerable theoretical attention, with a focus on state-dependency. Individuals differ consistently in a range of features or ‘states’, for example in morphology, physiology and even in aspects of their environment [18]. State-dependent behavioural models are therefore based on the fact that an individual's state influences the fitness costs and benefits of its behavioural decisions [18], [19]. As stable individual variation in growth rate has been reported in a variety of species with indeterminate growth [20], growth has also been suggested as a key factor in maintaining personality differences due to growth-mortality tradeoffs [20], [21]. Indeed, traits such as boldness, aggression and activity may correlate with higher growth rates, but these behaviours may also increase mortality through greater risk-taking [22]–[24]. As few empirical studies have tested predictions derived from state-dependent models, this is now needed to further our understanding of behavioural syndromes [25].

Individual variation in risk-taking was originally used to define boldness in animals [26], [27], which subsequently lead to a variety of interpretations on its measurement [28]. Boldness has been measured, for example, by response to threatening stimuli, novel objects or food sources, predator inspection, latency to emerge from cover and foraging under predation threat [*cf*. 28]. Although a consensus on the measurement of boldness is valuable for comparative purposes, a consideration of behaviours and related situations that would represent boldness in the species of interest is important [12], [13], [29]. While the biological significance of individual behavioural variation is increasingly recognised, the fundamental differences between functionally different species should not be overlooked. To interpret results from studies using different tests for measuring the same personality trait, the specific context and methods should be considered [13].

Pike, *Esox lucius*, exhibit considerable growth differences in their wild populations, with size dimorphism already apparent in young-of-the-year (YOY) [30], [31], thus making it a strong model to test size- and growth dependent personality. Pike is a solitary and cannibalistic predator species that does not live in groups during any stage of its life [32]. Cannibalism usually occurs between fish of different ages, but as considerable size variations occur within the same cohort, for example, among juveniles, individuals have been found to cannibalise on conspecifics 50–91% of their body size [33]–[35]. In addition, pike are vulnerable to attack from conspecifics of similar size while handling prey [36]. Due to the strong pressures from both intra- and interspecific predators on juvenile pike in their nursery habitats [30], [37], [38], an important trade-off has been suggested to occur between foraging activity in order to out-grow piscivores (due to piscivorous gape limitation) and anti-predator avoidance [39]. We therefore consider that a measure of foraging behaviour across a gradient of risks is an appropriate indicator of boldness in this solitary apex predator. Foraging under risk of predation has been used as a measure of boldness in several fish species [e.g. 40]–[43], but has recently been criticised as the behaviour measured might be motivated by hunger instead of boldness [12]. To overcome this, equal starvation periods prior to measurements of foraging behaviour are commonly used to ensure similar hunger levels among the test animals [44], [45].

Consequently, in the present study, we determine the presence of a behavioural syndrome in juvenile pike by estimating the repeatabilities of individual foraging behaviours through time and across risk situations, including at different stages over time, and its relationship to individual state (i.e. body mass) and growth rate. The following hypotheses were tested: (i) individuals exhibit stable behavioural syndromes, shown as repeatable foraging behaviour within and across risk situations; and (ii) in high-risk situations, larger-bodied individuals consistently forage at higher rates compared to smaller individuals, and so have higher growth rates in conditions of abundant food.

## Methods

### Ethics

The study was approved by an independent ethical review committee of Bournemouth University. The approval complied with the Home Office (HO) certificate in accordance with the UK Animals (Scientific Procedures) Act 1986. The study was conducted under the HO project licence number PPL 30/2626. Consent to collect the fish from the wild was granted by the Environment Agency of England and Wales. Electric fishing was carried out at the minimum power settings needed to incapacitate the fish and thus no adverse impact on other wildlife should have been experienced. In the laboratory, the fish were kept in isolation to prevent cannibalism. The pike were never in physical contact with either the similar or larger-sized pike during the competitor and predator trials respectively. All individuals resumed feeding within ten minutes after being returned to their holding tanks from the experimental tanks indicating low-stress levels. Individuals were observed daily for signs of disease or stress and were found to maintain a high level of feeding activity and health throughout the experimental period. At the end of the experimental period, all the fish (n = 55) were euthanized as stipulated by the HO project license with an overdose of the anaesthetic MS-222 followed by destruction of the brain. The fish were not released back into the wild due to legislative restrictions relating to fish stocking activities.

### Collection and housing of fish

YOY pike were captured in a tributary of the River Frome, Dorset (50°419 N; 2°119 W), between 15 and 20 May 2009 by hand netting. The fish were placed in 30 L buckets containing river water and air stones attached to a battery operated air pump to maintain oxygen levels before being immediately transported to aquarium facilities by car (transit time <40 min). After acclimatisation to the ambient laboratory temperature (16°C), the pike were individually placed in identical 25 L glass aquaria (32×30×26 cm) containing conditioned tap water, an air stone attached to an air pump for oxygenation and plastic plants for habitat enrichment. Three sides of the aquaria were covered with black plastic to prevent visual contact between individuals. Fish were fed *ad libitum* using *Gammarus* spp. for 10 days prior to the first experiment and continued between the experiments. A 14L∶10D photoperiod was maintained in the laboratory.

As predation by larger conspecifics (i.e. cannibalism) is a common threat to YOY pike [37], [46], to provide differential levels of predation risk in the experiments, age-1 pike (220–250 mm fork length) were captured from the same site on 22 May 2009 by electric fishing using a Smith-Root LR-24 backpack. These fish were kept individually in 60 L glass aquaria containing plant cover and were fed *ad libitum* with earthworms (*Dendrobaena veneta*).

### Experimental protocol

Thirty-four age-0 pike (initial mass W_i_ = 0.53±0.03 g, mean ± SE) were used as the focal fish in the experiments. This number of fish was chosen as previous work on repeated individual response experiments suggests a sample size of 30 will provide a moderate effect size and statistical power >0.8 [e.g. 41], . Individual consistency in foraging behaviour, as an indication of boldness, was measured repeatedly within and across three risk situations (treatments): i) no visual contact to other fish (control: no risk); ii) visual contact to a similar-sized age-0 stimulus pike (competitor: low risk); and iii) visual contact to larger-bodied age-1 stimulus pike (predator: high risk). Prior to each experiment, focal fish were starved for 24 h to ensure similar hunger levels among the individuals. Juvenile pike have high evacuation rate of ingesta, with 100% evacuation in 18–22 h and 24 h for juveniles of 0.15 g and 3 g respectively at 18°C [48]. The initial body masses of individuals studied here ranged from 0.23 to 1.21 g. Although the lower temperature in our experiments (16°C) may decrease the evacuation rates slightly, a starvation period of 24 hours is likely to be sufficient to standardise hunger levels. In addition, for the welfare of the fish, a starvation period of over 24 hours was not allowed within the HO project licence.

Each focal fish was removed from their keeping tank by scooping using a 0.5 L beaker and transferred to an experimental tank (30×20×20 cm) with a water depth of 10 cm. The water temperature and oxygen levels in the experimental tanks were the same as in the holding tanks. The fish were then acclimatised for 30 minutes with visual contact to the neighbouring tank, which, depending on the experimental treatment, was either empty (control), or contained a stimulus fish of age-0 (competitor) or age-1 (predator). Feeding behaviour was measured by filming their response to the subsequent introduction of ten live gammarids for 15 minutes. The fish were subsequently transferred back (by scooping) into their individual tanks. The control treatment was repeated six times, and the competitor and predator treatments were each repeated four times (repeats are from now referred to as trials), with this replication level satisfactory according to Bell *et al*. (2009) [49]. Each trial was completed in two days (between 9.00 and 18.00 h). Four to five days elapsed between trials and they were conducted in the following treatment-sequence: control, competitor and predator. Two additional control trials were carried out after this sequence had been repeated four times to increase the number of repeated measurements. All 14 trials were conducted between 1 June and 31 August 2009 (91 days). Large variation in growth rates during a similar time period has been reported in juvenile pike in the wild [31], [50] and in experimental conditions [38], [51].

The focal and competitor stimulus fish were matched for size within 5 mm. A minimum of ten different fish were used as competitor stimulus fish in one day, and a stimulus fish was not used twice in a row. Three predator stimulus pike were used, and they were kept in their experimental tanks throughout the experimental day. Focal fish were assigned randomly to the predator stimulus fish. No effect of time of day of the experiment or stimulus fish individual used (competitors or predators) were found on the behaviour of focal fish (ANOVA, p>0.05). At the end of the experimental period, final mass (W_f_) was measured for each individual. The specific growth rate (SGR) of each individual over the experimental period was calculated using the formula: SGR = [(ln(W_f_)−ln(W_i_))/*t*]×100, where *t* is the number of experiment days (n = 91).

### Video analysis

Video analysis enabled quantification of the following foraging behaviours: (i) latency of first prey attack (s); (ii) number of captured prey; (iii) number of unsuccessful attacks; and (iv) swimming activity (i.e. time spent moving). An unsuccessful attack was interpreted as when the captured prey escaped or was expelled. Individuals that did not attack prey were given latency times of 900 seconds so as not to remove the animals that were least likely to attack prey, but they were excluded from the variable ‘number of unsuccessful attacks’. All films were analysed by the same operator in randomised order.

### Statistical analysis

To evaluate whether the pike perceived the different risks we used Kruskal–Wallis tests to examine differences in the behavioural measures between treatments.

To test the first hypothesis, consistency of an individual's behaviour over time within situations and across situations (behavioural syndrome) was calculated as their repeatability (± SE and 95% confidence intervals) according to Nakagawa and Schielzeth (2010) [52]. Temporal stability of behavioural syndromes was tested by conducting four separate repeatability analyses using one trial of each treatment (control, competitor and predator) conducted closest in time. As one trial was conducted weekly (with 4–5 days apart) and in the same sequence (control, competitor and predator) the closest time between the control and competitor, and competitor and predator trials was 4 to 5 days and between control and predator trials 8 to 10 days. Repeatability was calculated using linear mixed-effects models for count data and generalised linear mixed-effects models for Gaussian data, both with individual ID fitted as the random effect and the behavioural variable as the dependent factor (rptR package in R) [52]. The number of captured prey and unsuccessful attacks constituted ‘count’ data and were analysed using the Poisson multiplicative overdispersion model fitted by PQL (penalised quasi-likelihood) estimation on the original scale. Latency to first attack and swimming activity were log-transformed and analysed for repeatability using the restricted maximum likelihood model. Both models use a randomisation procedure for significance tests. Only behavioural measures that were repeatable across time or situations were used in the subsequent analyses. In addition, between-situation correlations of the same behavioural measures and between different behavioural measures within-situations were investigated using Spearman's ranking test (r_s_). To test the second hypothesis, correlation analyses (r_s_) between repeatable behavioural measures and body mass (initial and final) and SGR were performed. All statistical analyses were conducted using R 2.12.1 [53]. To compare repeatability estimates, we compared effect sizes and the 95 percent confidence intervals in addition to determining whether the confidence intervals overlapped with zero rather than basing inferences purely on P-values [54]–[56].

## Results

The number of captured prey and swimming activity differed significantly between the three experimental situations (captured prey: K-W, Chi = 25.69, df = 2, P<0.001; swimming: K-W, Chi = 34.84, df = 2, P<0.001, Table 1). Higher numbers of captured prey and increased swimming activity were detected in the control and competition treatments compared to the predation treatments, suggesting adjusted responses according to risk levels.

**Table 1:** Mean behavioural measurements (± SE) of juvenile pike (n = 34) in each trial of the (a) control, (b) competitor and (c) predator treatment.

Treatment	Trial	Latency to attack (s)	No. of captured prey	No. of un-successful attacks	Swimming activity (s)
(a) Control	1	52.8±16.2	5.9±0.6	1.6±0.3	36.3±5.8
	2	152.2±26.0	6.7±0.5	1.1±0.2	36.0±3.3
	3	200.2±47.7	5.4±0.7	0.4±0.1	23.1±2.8
	4	222.5±46.9	3.9±0.6	0.4±0.1	36.5±4.4
	5	175.8±48.4	5.4±0.7	0.9±0.3	30.4±3.8
	6	207.3±46.1	3.9±0.7	0.7±0.2	39.0±7.3
(b) Competitor	1	111.3±22.4	3.3±0.4	0.6±0.1	18.8±1.9
	2	161.3±37.6	4.3±0.7	0.4±0.1	17.1±2.0
	3	214.4±46.0	4.2±0.7	0.7±0.2	29.4±4.5
	4	150.4±32.8	4.9±0.7	0.7±0.2	40.2±5.5
(c) Predator	1	80.2±36.1	1.9±0.3	1.0±0.3	9.3±1.9
	2	215.9±63.0	1.7±0.4	0.1±0.1	14.4±2.9
	3	332.6±64.2	1.3±0.4	0.3±0.2	16.0±2.8
	4	218.9±54.3	3.3±0.7	0.6±0.2	27.0±4.4

### Repeatability and stability of behavioural syndromes

Although repeatability was significant (P<0.05) for the latency to attack within the control and competitor treatments, the repeatability estimate was low (0.12), together with a CI ascending from 0 within the control (Table 2). The number of prey captured had significant P-values within all contexts, and although none of the CI overlapped with zero, the repeatability estimates and CI varied between contexts, with the competitor context having the highest effect size and CI. Repeatability analyses of behaviours across trials of each treatment conducted closest in time (i.e. temporal stability of behavioural syndrome), revealed significant P-values together with high effect sizes and CI for captured prey in trials 3 and 4 (Table 3. a–d). Latency to attack was also found to be significant within trials 4, whereas in trials 3, the CI started from 0. Across all experiments, the number of prey captured and latency to attack were significantly repeatable with high CI and effect sizes, whereas swimming activity, although having a significant p-value, had a CI starting from 0 (Table 3. e).

**Table 2:** The repeatabilities (R) of behavioural measures in juvenile pike (n = 34) within each experimental situation: (a) control (no risk), (b) competition (low risk), (c) predation (high risk).

	Behavioural measure	R	SE	95% CI	P
(a) control	Latency to attack prey	0.12	0.06	0.00 to 0.24	0.011
	Number of captured prey	0.19	0.08	0.05 to 0.35	0.001
	Number of unsuccessful attacks	0.10	0.09	0.00 to 0.32	0.094
	Swimming activity	0.07	0.05	0.00 to 0.09	0.086
(b) competition	Latency to attack prey	0.35	0.10	0.15 to 0.54	0.001
	Number of captured prey	0.44	0.13	0.18 to 0.68	0.001
	Number of unsuccessful attacks	0.00	0.10	0.00 to 0.32	0.660
	Swimming activity	0.07	0.07	0.00 to 0.23	0.192
(c) predation	Latency to attack prey	0.07	0.07	0.00 to 0.22	0.183
	Number of captured prey	0.21	0.13	0.03 to 0.53	0.026
	Number of unsuccessful attacks	0.00	0.29	0.00 to 0.85	0.788
	Swimming activity	0.08	0.07	0.00 to 0.25	0.138

Generalised linear mixed-effects and linear mixed-effects models (rptR package in R [33]) with fish identity fitted as random effect and the behavioural measure as dependent factor were used for calculating repeatabilities, standard errors, 95% confidence intervals (CIs) and P-values. Latency to prey attack and swimming activity were log-transformed to achieve normality.

**Table 3:** The repeatability (R) of behavioural measures in juvenile pike (n = 34) across context using one trial of each treatment conducted closest in time: (a) trials 1 (n = 3), (b) trials 2 (n = 3), (c) trials 3 (n = 3), (d) trials 4 (n = 3), and (e) all trials (n = 14).

Trials	Behavioural measure	R	SE	95% CI	P
(a)	Latency to attack prey	0.05	0.08	0.00 to 0.26	0.310
	Number of captured prey	0.10	0.11	0.00 to 0.36	0.171
	Number of unsuccessful attacks	0.21	0.18	0.00 to 0.65	0.174
	Swimming activity	0.00	0.07	0.00 to 0.23	0.473
(b)	Latency to attack prey	0.17	0.11	0.00 to 0.39	0.062
	Number of captured prey	0.19	0.14	0.00 to 0.49	0.085
	Number of unsuccessful attacks	0.00	0.27	0.00 to 0.86	0.809
	Swimming activity	0.13	0.10	0.00 to 0.36	0.143
(c)	Latency to attack prey	0.23	0.11	0.00 to 0.44	0.014
	Number of captured prey	0.41	0.16	0.12 to 0.73	0.003
	Number of unsuccessful attacks	0.27	0.25	0.00 to 0.86	0.339
	Swimming activity	0.11	0.10	0.00 to 0.33	0.147
(d)	Latency to attack prey	0.50	0.10	0.28 to 0.66	0.001
	Number of captured prey	0.70	0.12	0.43 to 0.89	0.001
	Number of unsuccessful attacks	0.00	0.18	0.00 to 0.55	0.948
	Swimming activity	0.00	0.06	0.00 to 0.21	0.608
(e)	Latency to first attack	0.18	0.05	0.09 to 0.28	0.001
	Number of captured prey	0.30	0.09	0.14 to 0.49	0.001
	Number of unsuccessful attacks	0.03	0.04	0.00 to 0.12	0.129
	Swimming activity	0.10	0.03	0.00 to 0.12	0.011

Generalised linear mixed-effects and linear mixed-effects models (rptR package in R, [33]) with fish identity fitted as random effect and the behavioural measure as dependent factor were used for calculating repeatabilities, standard errors, 95% confidence intervals (CIs) and P-values. Latency to prey attack and swimming activity were log-transformed to achieve normality.

**Figure 1:**
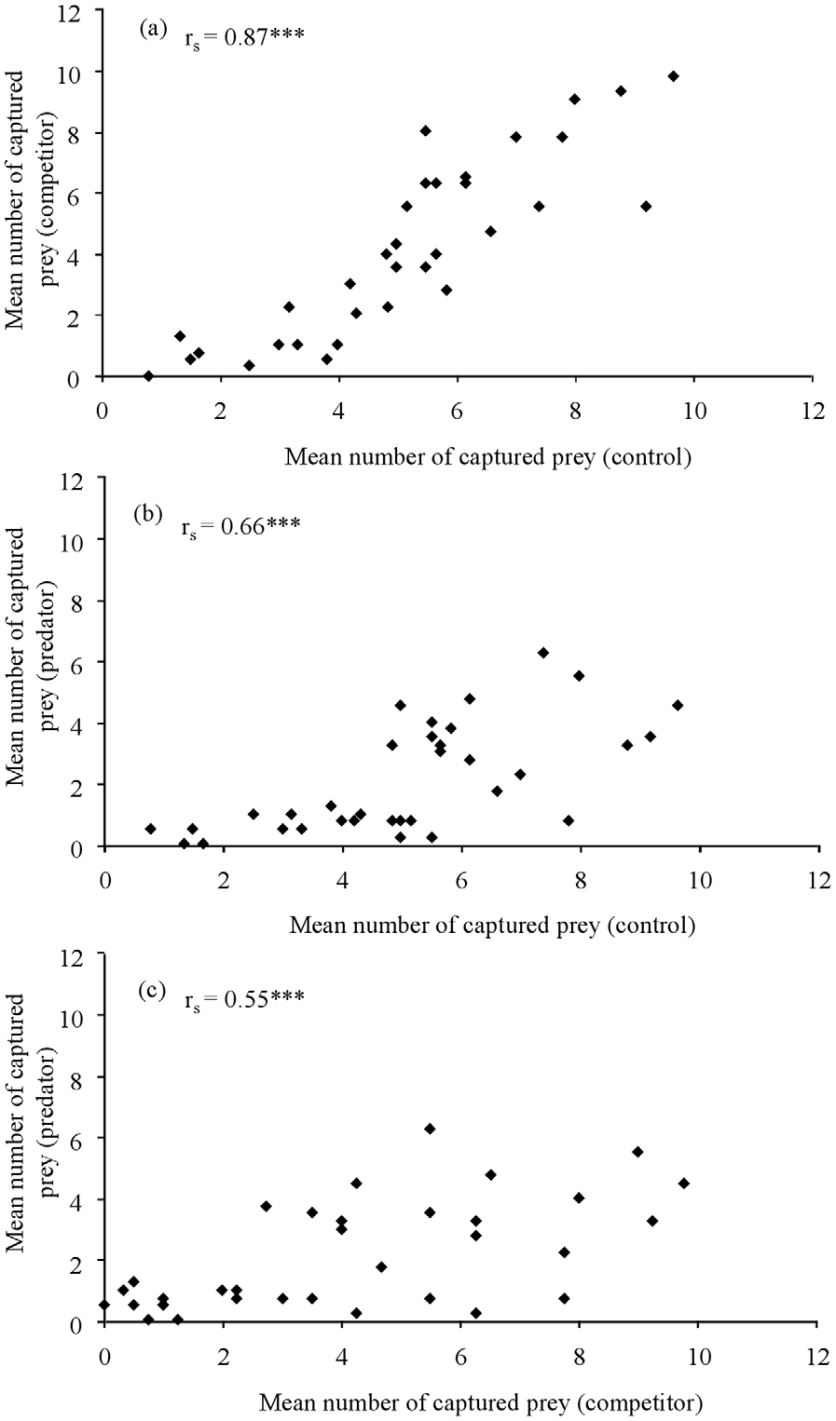
Mean number of prey captured per individual pike (n = 34) in the experimental treatments. (a) Control versus competitor, (b) control versus predator, (c) competitor versus predator treatments. Correlations were investigated using Spearman's ranking tests (rs,*** P<0.001).

Statistically significant correlations between treatments were found in the mean number of prey captured (Fig. 1), swimming activity (control and competitor, r_s_ = 0.43, n = 34, P<0.05; control and predator, r_s_ = 0.44, n = 34, P<0.05; and competitor and predator, r_s_ = 0.37, n = 34, P<0.05), and mean latency to attack (control and competitor, r_s_ = 0.51, n = 27, P<0.01; control and predator, r_s_ = 0.48, n = 30, P<0.01, whereas competitor and predator, r_s_ = 0.25, n = 34, P>0.05).

### State-dependent behaviours

**Figure 2:**
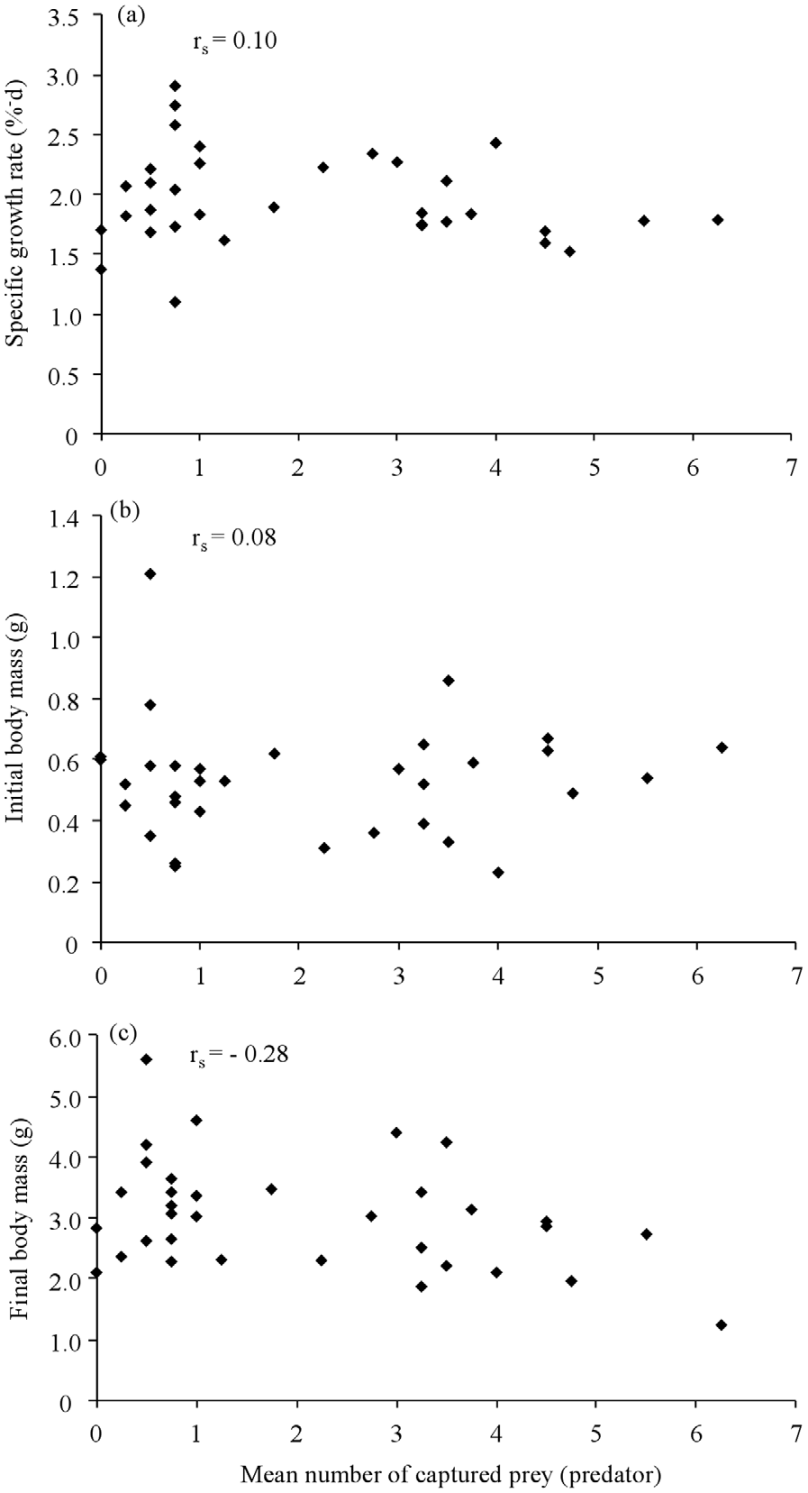
Mean number of prey captured in the predator treatment per individual pike (n = 34) and their metrics. (a) Specific growth rate, (b) initial body mass, (c) final body mass. Correlations were investigated using Spearman's ranking tests.

Neither initial mass, final mass, nor SGR correlated with any of the repeatable behavioural measures (i.e. number of captured prey, latency to prey attack and swimming activity) in any of the trials (all P>0.05; e.g. high risk, Fig. 2).

## Discussion

Consistent individual variation in foraging behavior across time and risk levels was evident in the experiments, suggesting the presence of a behavioural syndrome within a solitary predator of wild origin. Analyses of between-situation consistency over time revealed temporal instability of the behavioral syndrome over the experimental period and we found no evidence to support the state-dependent personality hypothesis.

The general decrease in feeding activity with increased threat found is consistent with Engström-Öst and Lehtiniemi (2004) who report that pike exhibit threat-sensitivity by decreasing prey attacks and swimming activity with the degree of predation risk [57]. Indeed, threat-sensitivity has been reported in a variety of vertebrate and invertebrate groups [58], and is obviously an important behavioural strategy as an under-estimation of risk may be fatal for the individual whereas an over-estimation may lead to unnecessary decreases in feeding activity. Consistent and significant individual variation in feeding activity over time, supported by between-situation correlations, indicates that some individuals were bolder in their foraging behaviour than others. As latency of prey attack, a common measure of boldness in fish [13], correlated significantly with the number of captured prey within all situations then prey capture was also considered an appropriate expression of boldness in the pike. Bold fish consistently continued to feed even during high predation risk (albeit at a lower rate), whilst others displayed consistently stronger risk-avoidance behaviour. The low but significant repeatabilities found here correspond to findings from a meta-analysis showing that significant behavioural repeatabilities often are low [49].

Many other studies of behavioural syndromes have conducted different experimental treatments using the same individuals on the same day [6], [41], [47]. However, when little time has elapsed in between observations of individual behaviour in different contexts, individual consistency across observations may be a consequence of the individual motivational state. As we conducted our treatments independently of each other with 4 to 5 days between trials, the behavioural consistency detected is more likely to reflect a relatively stable, unchanging aspect of the fish's personality.

Both temporal stability of the behavioural syndrome and consistency of individual behaviour that comprises the syndrome have been suggested to affect the strength of the selection force on the syndrome [6], [59]. Although consistency of individual behaviours was found across all trials, analyses of one trial of each situation separately exposed discrepancies with non-significant repeatabilities versus strong repeatabilities in the first two and last two repeats respectively. As individual consistency was found within each situation, the non-significant repeatability may be due to low between-individual variation across situations at first. Individual behavioural variation might increase due to experiential factors [59], so that individual behaviour might have become more distinct over time increasing the size of the variation between individuals.

Theoretically, individuals that are bolder and consistently take more risks to acquire food should grow faster [21], and through a positive feedback, also be larger in body size [60]. Growth differences have been found to persist even when individuals have been kept in isolation and fed *ad libitum* [61], [62], for example, in lizards [63], salamanders [64], turtles [65], and fish [66]–[68]. Here, however, the results revealed that despite some individuals repeatedly consuming more prey items during the experiments than others, these individuals did not achieve a higher growth rate during high food availability, nor was body mass related to the individual behaviour. The observed growth rates corresponded to the mean growth reported in their wild populations over a similar time scale during which size dimorphism has developed [31]. This suggests that individual growth differences do not occur as a consequence of individual behaviour alone but are likely to also be affected by a combination of spatial and temporal variation in the environment such as resource availability, competition level and/or predation pressure [69]–[71]. This is similar to the lack of correlation between behaviour and early growth rates found in steelhead fry (*Oncorhynchus mykiss*) when kept in a conventional hatchery-rearing environment [72]. On the other hand, in sibling dorada (*Brycon moorei*), kept in isolation and fed *ad libitum*, more aggressive individuals exhibited faster growth during the transition between food types [73]. In comparison, the pike in the present study were fed one food type throughout the experimental period, thus this might have been a factor reducing the potential for individual growth variation. The non-significant relationship between behaviour and body mass indicates that any differences between hunger levels of smaller and larger fish was unlikely to have affected their behaviour.

The present experiments characterised the presence of a behavioural syndrome in a solitary predator species, with individuals maintaining their foraging behaviour through time within the different situations. The ecological relevance of intraspecific variation including in behaviour is becoming increasingly evident [13], [14] and may be particularly important for populations of apex predators in their structuring effects on prey communities and food webs [74]. Indeed, the assumption that all individuals from predatory species have similar effects in structuring prey communities is being increasingly challenged by studies showing differences in foraging mode between species in the same habitats [75]. Such interspecific differences affect interactions between the predators and influence food web dynamics [76]. Thus, identifying behavioural differences at the individual level within a population may prove equally important in understanding the trophic dynamics in the ecosystems, and thus, there is a need to characterise appropriate behavioural syndromes in a wider range of species.
